# Newly Developed Electrochemiluminescence Based on Bipolar Electrochemistry for Multiplex Biosensing Applications: A Consolidated Review

**DOI:** 10.3390/bios13060666

**Published:** 2023-06-19

**Authors:** Christopher Mwanza, Shou-Nian Ding

**Affiliations:** 1 Jiangsu Province Hi-Tech Key Laboratory for Bio-medical Research, School of Chemistry and Chemical Engineering, Southeast University, Nanjing 211189, China; 2 Chemistry Department, University of Zambia, Lusaka 10101, Zambia

**Keywords:** bipolar electrodes, electrochemiluminescence, luminophores, multiplex assaying, biosensors

## Abstract

Recently, there has been an upsurge in the extent to which electrochemiluminescence (ECL) working in synergy with bipolar electrochemistry (BPE) is being applied in simple biosensing devices, especially in a clinical setup. The key objective of this particular write-up is to present a consolidated review of ECL-BPE, providing a three-dimensional perspective incorporating its strengths, weaknesses, limitations, and potential applications as a biosensing technique. The review encapsulates critical insights into the latest and novel developments in the field of ECL-BPE, including innovative electrode designs and newly developed, novel luminophores and co-reactants employed in ECL-BPE systems, along with challenges, such as optimization of the interelectrode distance, electrode miniaturization and electrode surface modification for enhancing sensitivity and selectivity. Moreover, this consolidated review will provide an overview of the latest, novel applications and advances made in this field with a bias toward multiplex biosensing based on the past five years of research. The studies reviewed herein, indicate that the technology is rapidly advancing at an outstanding purse and has an immense potential to revolutionize the general field of biosensing. This perspective aims to stimulate innovative ideas and inspire researchers alike to incorporate some elements of ECL-BPE into their studies, thereby steering this field into previously unexplored domains that may lead to unexpected, interesting discoveries. For instance, the application of ECL-BPE in other challenging and complex sample matrices such as hair for bioanalytical purposes is currently an unexplored area. Of great significance, a substantial fraction of the content in this review article is based on content from research articles published between the years 2018 and 2023.

## 1. Introduction

Analytical techniques based on electrochemiluminescence (ECL) and bipolar electrochemistry (BPE) [ECL-BPE] have in recent times emerged as indispensable tools in various fields, and of particular interest, in biosensing. Principally, biosensing involves the detection and collection of biological and biochemical information, which would be critical for diagnosing diseases [1] diseases, monitoring food and water quality [2,3,4,5], and environmental monitoring [6,7,8] among others. In clinical settings, diagnostic errors associated with some of the established methods in the detection of various biomarkers and other analytes call for serious concern. A couple of examples of events leading to diagnostic errors include misinterpretation of test results and failure to use an indicated or prescribed diagnostic test partly due to its complexity. The increase in complexity of analytical techniques comes with a considerable increase in the risk of technical errors during operation or malfunction may also increase. One other factor contributing to diagnostic errors in a clinical setting is the challenge of inaccurate results that occur as a result of insufficient and improper or poor preparation of samples including lack of proper instrument calibration. It is thus, of great essence to develop simplified methods of bioanalyte detection to aid the reduction in the incidences of false results especially in settings where resources including highly skilled analysts are limited and/or advanced analytical instruments may not be feasible or readily available.

A number of studies have shown that many of the widely employed biomarker assays used in medical facilities need constant improvement which may result from both low specificity and sensitivity associated with them [9]. Developing simple, cost-effective, and accurate methods for detecting bioanalytes with ease, while ensuring high sensitivity and selectivity is apparently crucial. Recently, ECL-BPE has emerged as a promising approach to detecting a variety of biomarkers and other analytes [10,11,12,13,14,15,16]. The excellent features and versatility of the ECL-BPE technique qualify it to be a valuable alternative biosensing tool due to its simplicity, high sensitivity, low background interference, and rapid result acquisition. Because of such remarkable features, ECL-BPE has found various applications, particularly in clinical diagnostics/bioanalysis [16,17,18,19]. Nonetheless, the technique has also been employed in food quality testing [20], environmental monitoring [21], and immunoassays [22] to mention but a few.

As the field of biosensing continues to evolve, research scientists are constantly seeking new methods for improved and efficient single and more advantageous, multiplex assaying of various chemical species of interest within a single run. Multiplex detection has become progressively important in reducing false positives and improving diagnostic accuracy [23]. ECL-BPE is one particular important analytical technique that has been and is continuously being explored for multiplex assaying, and here, we will highlight these multiplex assaying applications. At the onset, there are two configurations of BPE, i.e., open BPE (o-BPE) and closed BPE (c-BPE). While both systems have their strengths and limitations as shall be critically reviewed, recent research progress has focused on the applications of c-BPE for multiplex biosensing. Despite BPE being known for several decades, it has remained limited to only a few niche applications. However, in the past decade, novel and interesting approaches and applications of BPE working in synergy with ECL have emerged. This review article examines the application of ECL-BPE and for the most part, multiplex biosensing. It offers an in-depth analysis of the latest developments in ECL-BPE technology, covering aspects such as innovative electrode designs and recent advancements made in the development of novel ECL luminophores.

The review also discusses the existing challenges, including optimizing the interelectrode distance, the aspect of BPE miniaturization, and electrode surface modification, to improve sensitivity and selectivity, particularly for multiplex assaying. This write-up will not only present a three-dimensional perspective of ECL-BPE as an analytical tool, but its vast research content also highlights novel perspectives and identifies significant areas for future research and development. This perspective is especially relevant to the scientific community interested in exploring the latest advances in ECL-BPE and its appropriateness for a wide range of scientific fields, particularly in clinical settings. Furthermore, it adds value to the current literature as it not only discusses the strengths and limitations of ECL-BPE but also delves into its potential applications in multiplex biosensing, a topic that has not been adequately covered.

## 2. Methodology

**Figure 1:**
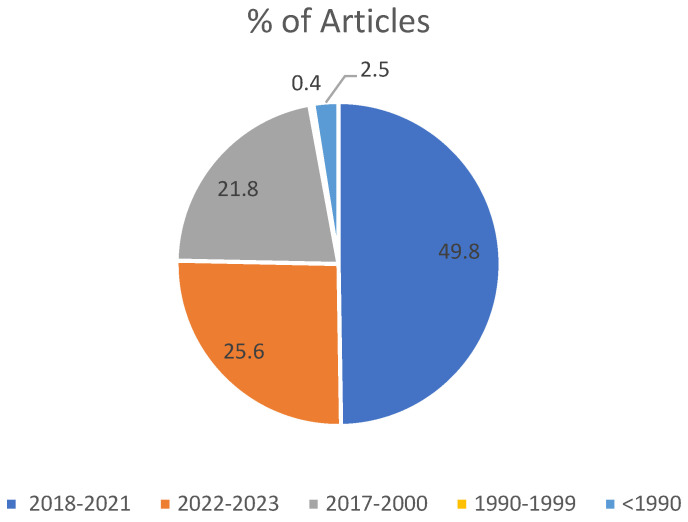
Article distribution based on publication year ranges.

A systematic and thorough approach to ensure accuracy of the content of this review paper was used to collect information. Firstly, when collecting the content, relevant keywords and concepts relating to the topic at hand were identified. These keywords and concepts aided in locating pertinent articles and papers from academic journals and databases. The majority of the scientific articles used here were obtained from Scopus, Web of Science, and PubMed. The titles, keywords, and abstract were first evaluated to determine the relevance of the article content. Any articles that lacked in their scope, the linkage between classical electrochemistry and BPE, closed and open bipolar electrodes, ECL-BPE and biosensing, the chemistry of luminophores and the concept of bipolar electrodes in synergy with ECL and their recent, novel applications in biosensing of a single bioanalyte or for multiplex assaying, were in the preliminary stage were not included. Much of the content in this review other than the typical theoretical aspect of both ECL and BPE is based on the past half a decade (between 2018 and 2023) of research work. The pie chart below (Figure 1) represents the percentage distribution of articles used in this paper, categorized by year range in which they were published. According to this chart, a total of 75.4% of the articles included in this review were based on articles published between the years 2018 to 2023, and 25.6% were exclusively based on publications made between 2022 and 2023.

## 3. Focus Questions

By and large, the following research questions guided this review:

(a) What are the strengths and limitations of closed bipolar electrochemistry (c-BPE) versus open bipolar electrochemistry (o-BPE) in biosensing applications?
(b) Which configuration of BPE is commonly employed in biosensing for improved multiplex assaying? For what reasons is it predominantly used?
(c) What are the available synthesis and functionalization strategies for bipolar electrodes, and how might they impact their suitability for biosensing applications in ECL-BPE, particularly in multiplex assays?
(d) Over the past half a decade of research, what advancements have been achieved in the development of novel ECL luminophores, and how have these advancements affected the ECL technique?
(e) During the past 5 years, what novel applications of ECL-BPE have emerged for multiplex assaying?
(f) With the focus on research conducted between 2018–2023, what are some of the challenges associated with using ECL-BPE for multiplex assays, and how have they been addressed, if at all?

## 4. Principles of Bipolar Electrochemistry

### 4.1. An Overview of Bipolar Electrochemistry

**Figure 2:**
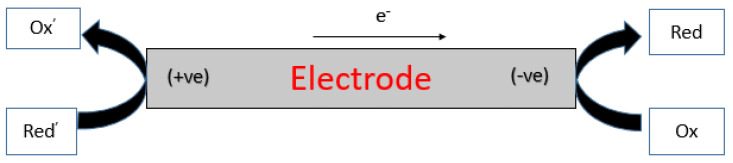
Schematic diagram of a substrate made of conducting.

Electrochemistry in general is a vibrant and versatile field that has seen increased attention due to its applications in various areas, including biosensors, chemical sensors, and electroanalytical chemistry. One of the most exciting aspects of electrochemistry is the existence of BPE designs that offers unique advantages in terms of sensitivity and selectivity. BPE exploits a working electrode being a bipolar electrode (BE, Figure 2) which is a single conductive object/substrate that drives redox reactions at its two ends [24]. The BE is remotely positioned in the cell where relevant analytes are collected [25]. In this technique, there are two distinct Faradaic reactions, that is, redox reactions, occurring at two ends of this conductive substrate (the BE) which has been polarized in an adequately high electric field [26].

As will be noted later, the BE can essentially be regarded as the heart of BPE. Worth noting is that BPE and traditional electrolysis are different but only slightly so. For example, the former is also a well-established “wireless technique” [27] that uses a conducting material immersed in an electrolyte solution and generates or induces asymmetric, electrochemical reactions on surfaces of two poles of a conductive object when a sufficient voltage bias is applied to the driving electrodes [28,29]. In BPE, the BE assumes both roles of an anode and a cathode and provides a physical medium for redox reactions to take place simultaneously [25]. The BE is by definition equipotential, however, the potential gradient that exists in the solution surrounding the BE will result in an inhomogeneous potential difference between the electrolyte and the BE. This potential difference varies along the BE’s main axis parallel to the electric field lines; this results in the polarization of the BE. When this is the case, one extremity of the BE will be the site of oxidation processes, whereas the opposite extremity will provide a surface for the reduction reaction [30,31]. The foregoing description of BPE is dissimilar to that of traditional electrochemistry where the two electrodes to wit, anode, and cathode are separated physically.

In the classical three-electrode electrochemistry system, the working electrode is the electrode of interest; depending on its polarization with regard to the electrolyte, either a reduction or an oxidation reaction takes place. On the contrary, this is not the case with BPE because as earlier stated, both reduction and oxidation take place simultaneously on the BE [30]. Moreover, BPE is also different from the traditional three-electrode system in the sense that the BPE set-up is much simpler [32] qualifying it for easier operation when also compared to other common analytical techniques. Nonetheless, in as much as at first glance, BPE would appear a bit different from normal electrochemical methods; but a thorough grasp of the underlying principles of the two techniques reveals that they are more similar than they are different. To be specific, in both BPE and normal electrochemistry, it is the interfacial potential difference that drives electrochemical reactions. The difference between the two is basically which side of the interface is being controlled, i.e., the solution or the electrode [33].

BPE has been a robust and reliable technique for some time now, and it has been used since the early 1970s for designing electrochemical reactors and batteries [34]. This was, of course, preceded by the groundbreaking research conducted by Fleischmann and co-workers who in 1969 described the concept of fluidized bed electrodes, where when a sufficient voltage is applied between two driving electrodes enables electrochemical reactions at conductive particles [35], i.e., BEs. To the best of our knowledge, there has been no other published work regarding this subject matter prior to the aforementioned, thus the findings emanating from Fleischmann and others’ investigations could be regarded as the foundation under which modern-day BPE is premised. Prior to the past two decades or so, BEs were largely referred to as fluidized bed electrodes and were used for designing industrial reactors meant for applications in processes such as electrosynthesis, and water splitting, and also for the purposes of increasing fuel cell performances [36]. Before being widely adopted for electroanalytical purposes, BPE had attracted great attention and had been used for many years in a variety of fields including battery technologies [37] concentration enrichment [38], separation [39] wastewater treatment [40], etc. In their research, Laws and others also investigated BPEs as a means of enriching and separating charged species electrokinetically where a single Au BPE was used to locally enrich several different charged markers in a single microchannel [39].

Whereas BPE can be applied for analytical and biosensing purposes, it still requires appropriate instrumentation and methods of detection to interpret the electrochemical signal that is produced at the BE. So in many cases for the purposes of providing an optical signal readout, BPE is usually used in combination with ECL, fluorescence, and anodic dissolution [41]. However, ECL possesses advantages over other forms of luminescence such as fluorescence because it does not require a source of light for photo-excitation; as a consequence, the instrumentation is quite simple thus, it is usually associated with low or zero background signal and allows optical detectors to be used at their maximum sensitivity [42,43,44]. In addition, there is also no backscattering [45]. The principles of BPE and ECL techniques will be discussed in detail in the forthcoming passages. Furthermore, what makes BPE when working in tandem with other detection techniques for multiplex assaying is because of its wireless capabilities which allow arbitrarily large arrays of BEs to be powered simultaneously in a very simple setup [46].

When BPE works in synergy with ECL, the sufficiently high voltage applied to the driving electrodes generates ECL at the anodic pole of each BE [47] which in turn facilitates a redox reaction where signal reporting is based on optical probes such as ECL. BPE, even though it is debatable, has also received tremendous attention in recent times due to the ease of miniaturization [48] as argued by advocates of BPE miniaturization. Because BPE has exceptionally unique properties coupled with the fact that it can be used for sensing, separations, and concentration enrichment in microelectrochemical systems [37]. Therefore, the technique is worth further exploring particularly for multiplex assaying of analytes including many different types of biomarkers in various sample matrices not only in the widely employed blood samples but also in non-invasive, yet complex samples such as hair. Overall, recent uses of bipolar electrochemistry concentrate on areas such as sensing, electrografting, electrodissolution, and electrodeposition, as applied to various fields including chemistry, biology, materials science, and device fabrication [49].

### 4.2. Fundamentals of Bipolar Electrodes

**Figure 3:**
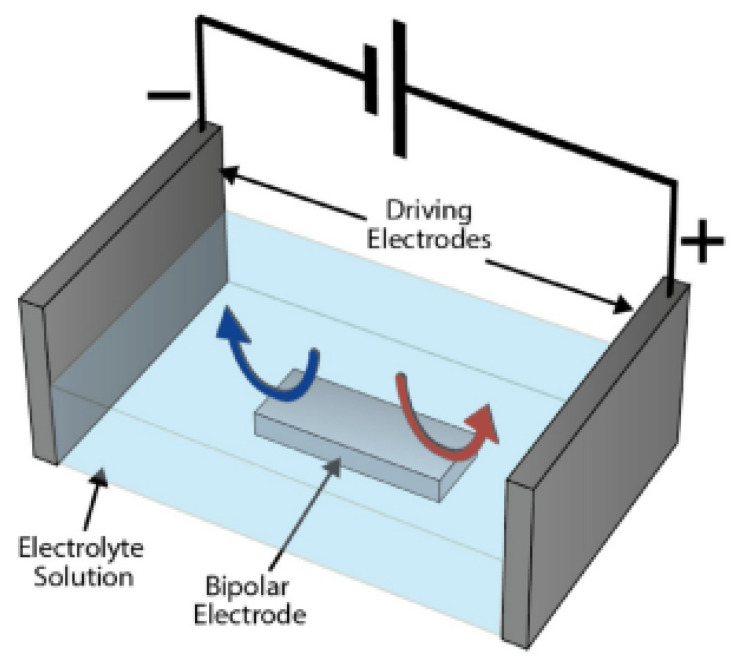
Schematic diagram of BPE with a conducting electrode placed in between two driving electrodes. The red arrow represents cathodic reactions whereas the blue one represents anodic reactions taking place at the ends of the BE. Adapted with permission from Ref. [33]. Copyright (2016) WILEY-VCH GmbH.

BPE, as aforesaid is essentially composed of a conductor (a BE) submerged in an electrolyte solution without direct connection to the external power source. A BE is an object made of a conductive material, such as a metal strip or bead, that facilitates electrically coupled Faradaic reactions at its opposing poles [50]. In terms of the experimental setup, the critical components of BPE include; two driving electrodes (feeder electrodes), an aqueous and inert dilute electrolytic solution, and an external power supply of uniform current applied across the solution [33,34]. The electrolytic solution can be a simple aqueous electrolyte or more exotic electrolytes such as ionic liquids [30]. The poles of a BE are oriented in the opposite polarity of the feeder electrodes [47]. Figure 3 illustrates a typical experimental configuration used for carrying out BPE. Here, the driving electrodes apply a uniform electric field across the electrolyte solution, and the resulting redox reactions at the BE are shown to occur at the anodic (blue arrow) and cathodic (red arrow) poles of the BPE. So the driving electrodes are employed to induce a potential difference across the solution of a bipolar electrochemical cell [50]. Basically, an electric field is induced across the electrolyte by an external bias of the feeder electrodes; as a result of the interaction between the solution and the BE, the BE develops what is known as floating potential and the potential gradient varies along its length. With this sufficient external voltage, a substantial potential difference may also be established between the ends of the BE. This potential that develops on the BE drives redox reactions where oxidation occurs at the anode end whereas reduction occurs on the other cathode end [48,51]. In essence, it is these Faradaic reactions taking place at the ends of the BEs that most BPE studies focus on [52].

The potential of the electrode (∆E_BPE_) floats to a value intermediate to that of the electrolyte it is in contact with. In a BPE cell, there is the development of interfacial potential differences with opposite signs at the ends of the BE. These interfacial potential differences are the anodic and cathodic overpotentials [33,50]. To maintain electroneutrality at the bipolar electrode, the electron production and consumption must be equal on both sides [37,53]. To estimate the potential across the BPE given as (∆E_BPE_), the following Equation (1) below can be used [54].

(1) $$∆E_{\mathrm{BPE}}=E_{\mathrm{tot}}\left[\frac{l_{\mathrm{BPE}}}{l_{0}}\right]$$

where E_tot_ is the voltage applied on the driving electrodes, l_BPE_ is the length of BPE, l_0_ is the distance between two driving electrodes. Basically, the parameters that control BPE processes include E_tot_, l_BPE_, and l_0_. Ultimately, ∆E_BPE_, being the driver of the redox reactions at the poles of the BE, is the main parameter taken into consideration for analyzing electrochemical processes in BEs [27].

**Figure 4:**
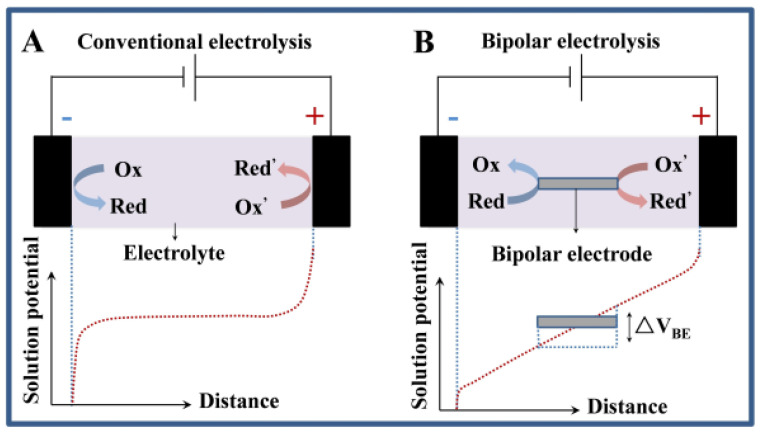
Comparison of electrolytic system types: (**A**) conventional electrolysis and (**B**) bipolar electrolysis. Adapted with permission from Ref. [29]. Copyright (2022) WILEY-VCH GmbH.

Figure 4 is an illustration of the comparison between conventional electrolysis systems and BPE. The (semi)conductor immersed inside an electrolytic solution is subjected to an electric field. These conditions split the interfacial nature of the BE into cathodic and anodic fields on which electrochemical reactions readily and simultaneously take place [29,55]. In BEs, potential decreases from the anodic pole to the cathodic pole, and oxidation reactions take place from the point where the anodic overpotential, ηa, is sufficient, whereas reduction reactions occur from the moment the cathodic overpotential, ηc, is sufficient [36].

### 4.3. Configurations of Bipolar Electrochemistry

**Figure 5:**
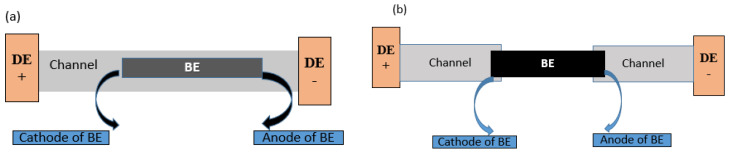
An illustration of an o-BPE (**a**) and c-BPE (**b**) systems. DE = driving electrode. Inspired by Ref. [57].

There are two types of BEs configurations that can be employed in BPE studies; these are open BPE (o-BPE) and closed BPE (c-BPE) systems [56] and a comparative schematic of the two systems is illustrated in Figure 5. These two systems differ in their design and operational characteristics. Both configurations have unique strengths and limitations that border on sensitivity, selectivity, reproducibility, cost-effectiveness, robustness, etc. Between the two, reports suggest that the o-BPE system was the first to be researched before c-BPE, which only appeared later [32].

In c-BPE, the bipolar electrodes’ two poles concurrently engage with two electrolyte solutions, each containing a driving electrode, which are physically segregated within separate chambers [51], and the BE provides the current path (electronic pathway without the presence of the ionic current path) between the two half cells [58] that is, the current flows in the two half cells through the BE only. This design ensures that the two poles on the BE are located in two separate solutions [59]. In c-BPE, the anode and cathode of the same BE are placed into different microchannels containing unalike solution electrolytes and so they are fluidically and chemically isolated [57]. In contrast, in the o-BPE configuration, the BE is located in one channel, i.e., located within a single electrolyte solution [51]. Basically, the microchannel itself acts as a conductor. In o-BPE as opposed to c-BPE, the electrochemical current passage through the BE is facilitated by the flow of electrons (electronic pathway) or may result from the mobility of ions in the electrolyte solution (ionic pathway) [34]. Generally, in electrochemistry, the application of a suitable external driving voltage between the feeder electrodes generates a current that flows either via ionic or electronic pathways [56,60]; the latter current is the one that is relevant to BPE and to exploit this current efficiently, a BE with a conductivity higher than that of the electrolyte solution is used [34].

### 4.4. Closed vs. Open Bipolar Electrodes: A Comprehensive Review of Their Limitations and Advantages

Two different configurations of BPE, c-BPE and o-BPE possess different strengths and limitations. To start with, it is argued that c-BPE possesses some exclusive merits, notably in the area of detection. For instance, it has been reported that the c-BPE configuration has the distinctive ability to separate the reagents for generating detection signals such as ECL [61] and fluorescence from the targets of detection, which is actually conducive to reducing the background signals and interferences [56]. c-BPE also requires less driving potential to drive the Faraday reaction and is characterized by higher electron transfer efficiency (which is 100% in theory) in comparison to o-BPE [62]. Faraday reactions basically are electrolytic reactions that play a pivotal role in BPE and involve the oxidation or reduction at surfaces of a BE. Typically, they comprise the movement of electrons from one electrode to the other. Oppositely directed Faradaic reactions (reduction/oxidation) may be caused at the cathodic and anodic sides of the BE due to the potential difference between it and the electrolyte [63]. Furthermore, the c-BPE system offers somewhat a novel level of suppleness in respect of experimental design because a variety of conducting solutions with a diverse chemical constitution can be implemented in the very two independent compartments. The experimental conditions (pH, temperature, and light exposure) can also be manipulated independently to favor desired reactions [51]. These distinguished qualities of c-BPE have seen interest spike in its applications in the past decade as evidenced by the enormous numbers of scientists using c–BPE in their research works as shown in Table 1. For example, it was utilized in the investigation of electrical coupling between the BE photosystems 1 and 2 [64] and is of great interest because of its relation to this article; c-BPE was utilized in the development of an electrochromic sensor for multiplex detection of a variety of metabolites [65]. The ability to perform multiplex detection in this research was achieved by using a c-BPE electrochromic detector which was modified by integrating three sets of detection chemistries into one sensing device and detection of the analytes viz. glucose, lactate, and uric acid simultaneously demonstrated. In addition, another research group [66] demonstrated side-by-side the difference between o-BPE and c-BPE. In this investigation, it was deduced that excellent spatial resolution and specific surface functionalization of the c-BPE-ECL approach made it suitable for constructing multiplex imaging platforms with improved sensitivity. In addition, an ECL based on c-BPE was also used in the visual screening of *Salmonella* typhimurium with a very low limit of detection [67]. So, modifying c-BPE may also enhance the detection sensitivity, and selectivity accuracy of multiplex assaying.

**Figure 6:**
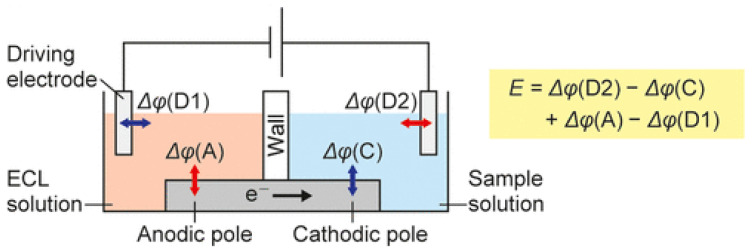
Schematic of c-BPE with two separate solutions. (Δφ(D1), Δφ(D2), Δφ(C), and Δφ(A) represent the potentials of the driving electrodes 1 and 2). Reprinted with permission from Ref. [75]. Copyright (2022) ACS publications.

Some studies suggest that in the o-BPE system, the detection relies on ECL or visual metal electro-dissolution [42] and this is because of the difficulty in measuring the direct current that flows through the BE [61,68]. On the contrary, in the c-BPE configuration where the two poles of the BE are in two individual compartments, it is contended that measuring the electric current is somewhat easier [69]. c-BPEs have several other advantages, including a high selectivity of the reaction taking place, low background current, and the ability to eliminate interference from other electroactive species. However, other limitations of c-BPE also include their relatively low sensitivity and ability to detect only specific ions or analytes [70]. To add on, based on recent research publications, o-BEs are generally preferable over c-BEs in biosensor applications due to their high sensitivity and versatility in detecting multiple analytes. c-BEs, on the other hand, are usually useful in ion selective electrode applications [71], potentiometric sensors [72], and detecting low levels of heavy metal ions in the environment [73]. What is also worth noting is that o-BPE systems are advantageous because of their capability to incorporate with comparable ease, a large number of BEs [74]. However, the shortcoming is that in this o-BPE system, the output signal could easily be influenced by the electroactive chemical species since they are present in the same solution. On the other hand, this limitation is avoided in c-BPE because the anodic and cathodic poles are immersed in different solutions in separate compartments. Figure 6, which is a schematic for c-BPE can be referred to for more details [75]. Nonetheless, as a consequence of the presence of the separating wall between the two compartments, some studies that have employed the c-BPE configuration for multiplexing have only been able to incorporate a limited number of BEs in the system [76,77,78]. In the o-BPE configuration, however, this may not be the case because the incorporation of a limitless number of BEs is possible.

For the most part, however, recent research has predominantly focused on the use of c-BPE in the development of ECL-based multiplex biosensors. The c-BPE configuration possesses several advantages over o-BPE, including improved reliability and reproducibility, reduced electrode fouling, and enhanced kinetic reversibility. c-BPE is especially compatible with fabricating sensors based on ECL. ECL is a process that involves the conversion of electrical energy into luminescent output. By separating the sensing and reporting reactions at opposite ends of the BPE, the electroactive substances generated in the biosensing system can be isolated from the ECL reaction, thereby enhancing the flexibility of the biosensing approach used [78]. These advantageous features make closed BEs ideal for use in the development of high-performance electrochemical biosensors. Over the last ten years or so, researchers have placed more emphasis on the use of c-BPE within the analytical field as compared to o-BPE [62]. Overall, the use of c-BPEs in ECL-based biosensors has enabled the accurate and sensitive detection of multiple analytes in a single assay, making it a highly promising technique for multiplex biosensing applications. As will be observed later, several studies have demonstrated the effectiveness of c-BPE in the development of multiplex biosensors. Nonetheless, researchers may still carefully evaluate the advantages and limitations of both systems in terms of targeted application requirements. Further research is required to enhance and optimize the performance of bipolar electrodes for different biosensing applications.

### 4.5. Bipolar Electrode Miniaturization: Uncovering the Opportunities and Challenges

As noted in the preceding sections, one of the potential advantages associated with BPE is its ability to be miniaturized: miniaturized BPE has been used in various devices for instance in microelectronic devices and point-of-care devices [25,57,79], and it also has the potential to be incorporated in environmental monitoring systems, among other applications. Miniaturized devices also go by the following names; microfluidic devices, lab-on-a-chip, and micro total analysis systems [80]. Generally, miniaturization is associated with cost-effectiveness in terms of materials needed for electrode fabrication along with small volumes of sample, fast response, precision, multiplex operation, and rapid results acquisition [81,82]. The reduction in sample volume and cost, accompanied by the possibility of automation, can provide significant benefits in the point-of-care and clinical settings, particularly in developing countries where both human and financial resources may be limited. The development of microfluidic chips (miniature devices) has also greatly contributed to the simplification of the operation of BPE. Microfluidic devices offer cost, reagent, and time efficiencies in comparison to traditional devices, exhibiting their strength in biological sample detection and the development of portable detection tools [78]. It is no doubt that the miniaturization of bipolar electrodes is a viable concept.

However, despite the potential benefits, miniaturizing BPE presents numerous challenges. Some research scientists have highlighted a couple of limitations regarding BPE miniaturization. For instance, it is argued that despite BPE being a well-established technique, its inherent inadequacy is generally related to the length of the BE: the shorter it is, the larger the applied potential required to have polarized and consequently induce redox reactions at its two poles [34,47]. This limitation constitutes a very important drawback for nanometric and micrometric BEs as high potentials in the order of tens of kV or more is required to successfully achieve their polarization [83]. Therefore as observed by another independent researcher [26], it would be problematic to apply an electric field to attain polarization of a micro- or nonmetric metallic particle acting as a BE in a living cell to conduct wireless biosensing, for example. Other challenges include issues to do with scalability and sensitivity. Miniaturization comes with increasing complexity and additional effects, showing deviations from macroscopic models, have been reported. These effects can occur due to some unexpected behaviors at the nanoscale, which can be summarized as “nano-effects” or because of additional interfaces causing confinement that affects the electrochemical process, such as “confinement effects” [84]. To address these challenges, researchers have proposed various strategies, such as optimizing the interelectrode distance and selecting appropriate materials for the electrodes to enhance the sensitivity [85]. These approaches, alongside further research and development, provide promising opportunities to overcome the challenges associated with the miniaturization of BEs. The microfluidic format has also been reported to face two major obstacles that is, limited sensitivity attributed to the tiny sample volumes employed and the cost of microchip production [80]. However, the complex microchip system can be replaced with a cost-effective textile substrate featuring inbuilt microchannels built in the mesh of its fibers. Additionally, integrating this textile-based microfluidics with electrophoresis and BPE can lead to considerable enhancements in solute detection, as the analytes of interest are concentrated and focused [86,87].

All in all, two interesting properties of BPE that have justified the attention it has garnered in the past years of research are due to two noteworthy characteristics: (a) its wireless nature that eases electrochemical sensing and supports high throughput analysis; (b) the gradient potential distribution on the surface of the electrode is a useful tool for preparing gradient surfaces and materials. These important characteristics make BPE very suitable for further modification and analytical applications even on a micro/nanoscale surface [29]. In conclusion, the miniaturization of ECL based on BPE is a promising and rapidly growing field. Despite the challenges, the technique offers great opportunities for developing analytical devices for point-of-care applications, and the efficient monitoring of biological and bioanalytical systems.

### 4.6. Synthesis and Functionalization Strategies of BEs for Biosensing

The fabrication of BEs should be adapted according to their intended application in different fields by utilizing an assortment of materials and dimensions. Polyethylene, glass, and polymethyl methacrylate are commonly utilized in their preparation due to their transparency [27]. The exact design and composition of the BE substrate will be dependent on the specific application and desired properties. Various materials can be employed as driving electrodes in BPE, such as metallic substances such as Pt, Ag (or Ag/AgCl), Au, and stainless steel [88,89,90]. The synthesis and functionalization strategies of BE play a critical role in determining their corresponding performance in biosensing. In terms of synthesis techniques, electrodeposition is a common method used for the synthesis of both carbon-based materials and metal-based nanoparticles. Electrodeposition allows the control of size, shape, and chemical reactivity of the nanoparticles formed [91,92]. For example, carbon nanotubes can be synthesized by chemical vapor deposition (CVD) using a variety of catalysts [93]. Thin films of polyimide can be deposited using spin coating [94]. BE fabrication can also be achieved using several materials such as glassy carbon [95]. It is well-recognized that carbon-based materials such as glassy carbon, graphite, and carbon nanotubes are desirable due to their distinct advantages, such as electrical conductivity, biocompatibility, low cost, and straightforward fabrication [96,97,98]. As an example, our research group [99] also fabricated an inexpensive and disposable sensing platform for detecting H_2_O_2_ using the pencil core as the BE. The design of BEs for ECL-based biosensing requires a careful choice of materials used in its fabrication, synthesis techniques, and functionalization strategies.

Functionalization of the BE is one way in which the range of different analyte detection using the BPE technique can be improved. Functionalization of materials involves modifying their surface chemistry to add novel features, capabilities, or properties [100]. In another study, BE was fabricated by microchip manufacturing technology; here, it was observed that the BE mainly exhibited the electrochemical behaviors of one pole in a one-half cell, but was influenced by the redox reaction in the other half-cell [56] As observed so far, it is very possible to fabricate hybrid conducting materials with a range of functionalities both at the macro- and nanoscale [36] and this is quite interesting from the multiplex biosensing viewpoint. Another study [57] was conducted to create an ECL system that is both sensitive and portable, using laser-induced graphene-BE for biosensing vitamin B-12. The device was made using polyimide (PI) material and the BE system was fabricated by subjecting the PI sheet to a carbon dioxide laser. PI offers several benefits such as high dependability, greater stability, a one-step manufacturing process, and swift prototyping [101]. Several biofunctionalization strategies can be used to enhance BPEs’ properties and allow for the detection of specific analytes. Biofunctionalization strategies using antibodies, DNA/aptamers, and redox enzyme immobilization have been widely used to promote analyte recognition [102] for multiplex biosensing applications, for example. The surface of BEs can be modified to promote analyte recognition and improve their electrochemical properties by biofunctionalizing them with biocompatible and biorecognition molecules. As a significant example, nano-bovine serum albumin (nano-BSA) has shown great potential as a biofunctionalization agent due to its unique structural and chemical properties. The high surface area-to-volume ratio of the nano-BSA enables more binding sites for biomolecules [103,104] while its stability in various buffer solutions allows for long-term use in biosensing applications, Additionally, its biocompatibility and low immunogenicity make it an excellent choice for use in various biological applications, including biosensors. For instance, in one research, nano-bovine serum albumin was utilized to biofunctionalize a graphene interface to create a highly sensitive multiplex assay for detecting cancer biomarkers [105]. In another study, a glassy carbon electrode’s surface was fabricated with high luminescent polydopamine nanospheres and chitosan using the layer-by-layer assembly method for detecting Mucin-1 [106]. Stimulatingly, however, one study [107] developed a single electrode electrochemical system (SEES) for multiplex assaying and they were successful. The SEES which utilizes only one electrode has a microelectrochemical cell built with a hole in an insulating self-adhesive plastic film for a single experiment and multiple holes for multiplex experiments. When an external voltage is applied to both ends of the single electrode, it generates a potential difference between the two ends of the microelectrochemical cell and triggers Faradaic reactions. The system generates ECL reactions through a potential difference induced by the electrode’s resistance [108]. In comparison to traditional BPE, this SEES system possesses a couple of inherent advantages such as being further simplified and more cost-effective especially for high throughput analysis since only a single electrode is in use. The system is comparatively economical because it does not involve complicated and costly fabrication procedures of the electrode arrays. Further, the system is also devoid of ECL background problems from the driving electrode in the BPE system. However, this system is yet to be extensively explored.

In general, the use of a substrate made of conducting material as a BE offers significant advantages for electrochemical applications. The BE design further improves the efficiency and versatility of BE by allowing for the simultaneous use of both sides of the electrode. Further research and development in this area will likely lead to new and innovative uses for this technology. In summary, BPE is a promising technique that has a wide range of applications in various fields. In the field of biosensing, BPE has the potential to improve the sensitivity and selectivity of electrochemical sensors by generating more stable, reproducible, and controllable signals. Because of being wireless and lack any connection to an external device, the electrochemical reactions occurring at one pole related to detection are usually reported through ECL on the other BE [75]. Currently, ECL is the most prominent reporting strategy for analytical BPE. BPE allows for the spatial segregation of sensing and reporting electrodes, and ECL offers an easy-to-read, sensitive visual display [56]. Hence, by incorporating BPE with other techniques such as ECL, the sensitivity and detection limit of biosensors can be further enhanced. As it shall be noted later, the combination of BPE with ECL offers exciting new opportunities for enhancing the sensitivity and specificity of multiplex assaying. The synergy between these two techniques is indubitably essential for developing highly sensitive, real-time, and reliable biosensing systems.

## 5. Fundamentals of Electrochemiluminescence: A Summary

Electrogenerated chemiluminescence or electrochemiluminescence is a form of chemiluminescence (CL) process generated as a result of redox reactions occurring at or near the surface of electrodes; it is a process by which electrical energy is converted into light (radiative energy) [109,110]. ECL and CL in turn are both forms of luminescence—which is a process of light generation without using a excitation light source [111]. However, ECL has an inherent preeminence including enhanced temporal or spatial controllability. The ECL process is switchable concomitantly with the applied electrolytic potential; hence, electrochemical parameters can be modified accordingly to improve its performance [112]. The emitted light is typically the analytical signal [44]. Notably, ECL is an amalgamation process involving both electrochemistry and CL. ECL is an excellent combination of spectroscopy and electrochemistry [113].

As a consequence of its integration of electrochemical with spectroscopic methods, ECL also exhibits the following valuable and exceptional characteristics: high spatial resolution, low background signal, high sensitivity and specificity, high throughput, wide dynamic range, and simple instrumentation designs [114,115]. The lack of an external excitation light source and having near-zero background has immensely contributed to the high sensitivity associated with ECL and has an apparent advantage in both biosensing and imaging applications [116] coupled with insensitivity to matrix effects as another exceptional additional beneficial characteristic [117]. The ECL-based biosensors combine the advantageous features of both highly sensitive ECL analysis techniques and the high selectivity of biological recognition of bioanalytes [118]. Furthermore, electrochemical techniques provide a limit of detection (LOD) in the micromolar range, whereas ECL offers greater precision, with LOD closer to the picomolar range [119] making it a technique of choice for bioanalysis [120] in the medical prognosis and diagnosis, food control and environment [116]. To add on, unsophisticated photonic detectors that have been designed possess the capabilities to detect ECL emission [42] including readers built on a cellphone platform [121]. In consequence, the ECL technique has certainly proven to be superior to conventional spectroscopic analytical techniques [122,123,124]. Broadly speaking, the evolution of ECL is significantly impacted by three fundamental factors: luminophores (also known as emitters or fluorophores), co-reactants, and electrodes. In the upcoming section, we present a brief summary of recent advances made in these three significant components of the ECL systems.

### 5.1. Recent Advances in ECL Luminophores

Over the past decade and beyond, there have been numerous ECL luminophores that have been developed for use in biosensors. Recent advances in novel ECL luminophores have also led to the development of new classes of luminophores that exhibit specific properties and that can be used for a variety of applications. Used as signal probes, luminophores are a major constituent of an ECL system. One crucial step towards developing and utilizing ECL involves the designing, identification, and selection of appropriate luminophores that can serve as ECL emitters [123]. ECL biosensing utilizes various types of ECL reagents, and among them, the three significant ECL systems are nanomaterials (NMs), inorganic metallic/organometallic complexes, and organic polyaromatic hydrocarbon compounds [43,125]. Two optimal ECL reporting reagents for the BPE application are Ru(bpy)_3_^2+^ and luminol due to their high luminous efficacy and intensity, which are particularly useful for visual imaging [46]. The ECL luminophores properties of Ru(bpy)_3_^2+^ were first reported in the year 1972 [126]. ECL luminophores based on Ru(bpy)_3_^2+^ and its derivatives have been widely employed in the fabrication of ECL-based biosensors [127]. However, both of the aforementioned predominant ECL reagents are only excited at positive potentials, so they only occur at the anodic pole of the BPE. This implies that, due to the charge balance of the BPE poles, targets would be on the cathodic pole, where as expected, the reduction reaction occurs. It becomes challenging to detect the target oxidation reaction directly based on the above BPE-ECL sensing strategy. Consequently, this limitation restricts the ECL-BPE sensing strategy’s further application [128]. The foregoing has necessitated the need to develop alternative, novel ECL luminophores with superior properties, if possible.

There is a high expectation for the development of luminophores capable of near-infrared (NIR) ECL emission, with potential candidates including tetraphenylethylene [129], and Ag–Ga–In–S (AGIS) nanocrystal [130]. The field of biological sensing and imaging has remained continuously interested in NIR emission due to its exceptional benefits. These include lower background interference, reduced photochemical damage, and superior tissue penetration ability [131]. Nevertheless, the progress in this field remains limited, with only a few luminophores developed that possess an ECL emission wavelength surpassing 800 nm in an aqueous medium. One such example is the recently discovered Co^2+^-doped CdTe nanocrystals, which exhibit a maximum ECL emission at approximately 815 nm [132] compared to CdTe with an emission of λmax = 782 nm [133].

NIR luminophores are regarded as novel and are based on quantum dots (QDs), polymer dots, organic nanoparticles (NPs), and metal nanoclusters and they have been arousing the enthusiasm of a number of researchers [134]. Attributable to the quantum confinement effect, QDs have been established to be an essential sub-category of luminophores. They have exclusive optical, chemical, and electronic properties owing to their size being close to the exciton Bohr radius [135]. Compared to other common luminophores such as luminol and Ru(bpy)_3_^2+^ quantum dots (QDs) have several superior qualities. Firstly, QDs have exceptional nanostructures and can emit multiple colors by modifying their composition, size, and shape. Secondly, QDs are well-suited for the design of ECL sensors due to the availability of advanced surface modification and conjugation technologies. Thirdly, QD-based ECL sensor systems can perform multiple sensing strategies and assay applications, including multiplex assays. QD-based ECL systems can also use S_2_O_8_^2−^ as an innovative co-reactant [136].

Newly developed ECL luminophores based on QDs functioning as novel ECL emitters such as MoS_2_ QDs [137,138] and organic nanocrystals such as perylene nanocrystals [139] are currently being intensely explored with the requirements of excellent optical properties and environmental friendliness [140]. During the past decades, organic luminophores have widely been employed in a range of different areas of applications including in biosensors owing to their inexpensive production, structural tailorability, and excellent optoelectronic characteristics [139]. For example, on account of their superb properties, such as facile modification, well-defined molecular structure, and good biocompatibility, small organic molecules are examples of organic luminophores that have in recent times sparked curiosity among analytical chemistry researchers [140].

In recent years, carbon NMs such as carbon quantum dots (CQDs) have attracted significant attention as promising ECL luminophores. CQDs have low toxicity, excellent biocompatibility, and chemical inertness, and tunable photoluminescence properties [141]. These characteristics make them a promising alternative to heavy metal-based quantum dots for ECL. QDs based on carbon and graphene which are classified as zero-dimensional nanomaterials have been attracting an escalating interest in biosensing and imaging [142]. While significant research has been conducted on CQDs, there are still some shortcomings that require attention. Specifically, CQDs generated from fine carbon structures such as graphene or carbon nanotubes, or traditional chemicals, may not be entirely benign [143]. Regardless, CQDs are currently being considered to be the next generation of alternative and less harmful NMs and it has been established that they are also favorable materials for a multitude of interesting applications including imaging and biosensing [144] because they do not only demonstrate exceptional luminescence abilities but also possess the advantages of easy labeling, low cost for synthesis/fabrication, unique optical properties, high affinity and specificity to targets [145,146]. In the year 2018, a novel ECL NM was also synthesized by dual luminophores perylenetetracarboxylic acid and CQDs and used for carcinoembryonic antigen detection. The assay demonstrated high sensitivity and low limits of detection [147]. Elsewhere [148], an ECL system based on carbon/graphene QDs and sulfite SO_3_^2−^ (as a novel co-reactant) has also been reported. Novel ECL luminophores based on the europium metal–organic framework (Eu-MOF) have recently been developed using a specific 2,4-bis(3,5-dicarboxyphenylamino)-6-oltriazine (H4BDPO) ligand with acid–base buffering properties. The Eu-MOF-based biosensors designed using this luminophore showed sensitive and effective detection of trenbolone analyte. Furthermore, the biosensors were capable of operating under a broad range of pH levels [149]. In the past few years, it has been established that MOFs are superior to other luminophores in terms of their high mass transfer capability within their pore channels, leading to faster reactivity. This quality of MOFs facilitates the enhancement of performance in ECL sensors [125].

ECL luminophores based on transition metal complexes and clusters are also traditionally employed as ECL luminophores [150]. Ru(bpy)_3_^2+^ complexes exhibit appealing biological, catalytic, electronic, and optical properties; in addition, they have also found an exclusive niche in the area of biosensing. However, while it has excellent ECL performances, it can be expensive to synthesize and difficult to immobilize onto the surfaces of the electrodes [151]. The emission of Ru(bpy)_3_^2+^, and its derivatives also occurs within a restricted wavelength range of approximately 600 nm [152]. However, ECL luminophores such as QDs and organometallic complexes, such as Ru(bpy)_3_^2+^ can be difficult to produce or have lower ECL emission efficiency due to their π–π stacking [153], which limits their practical applications. Thus, researchers are now focusing on MOFs that use conventional organic ECL emitters as ligands and incorporate metal to enhance ECL emission, making these MOFs a topic of significant interest [154]. Additionally, a new kind of high luminescent polydopamine nanospheres was developed using potassium persulfate as a co-reactant for Mucin 1 detection. This ECL luminophore belongs to the organic luminophore category and displayed great sensitivity and specificity [106]. Moreover, in order to enhance the performance of ECL biosensors in detecting a range of analytes, other various NM-based luminophores have been extensively utilized. These NMs comprise ordered mesoporous silica–metal–organic frameworks, covalent organic frameworks, and metal–polydopamine frameworks, among others. These materials have been effectively employed in ECL biosensors for detecting heavy metal ions, small molecules, proteins, and nucleic acids, among other substances [155]. Table 1 presents a summary of some more novel luminophores that have been recently developed as a result of research over the past five years.

**Table 1:** Summary of partial studies on novel ECL luminophores and respective co-reactants based on past 5 years of research.

ECL Luminophore	Luminophore Class	Main Co-Reactant	Notable Observations	Ref
La^3+^-BTC MOFs	Inorganic-organic hybrid	-	Prepared as the highly active reactor.	[125]
Tr-HOFs	Inorganic metallic/organometallic complexes	-	LoD: 0.28 nM; label-free ECL biosensor; highly improved ECL efficiency that that of Ru(bpy)_3_^2+^	[154]
Pc gadolinium complex	Metal complex	* In_2_O_3_/ZnIn_2_S_4_	Co-reactant possesses unique hollow structure-related advantages.	[156]
Zn-MOFs	Inorganic–organic hybrid	-	A co-reactant-free ratiometric ECL biosensor	[157]
CD_P_s and oxidized CD_P_s	Organic NMs	-	Synthesized by pyrolysis; o-CD_P_s ECL performances better than CD_P_s; Ultrasensitive and high selective benign sensor.	[158]
Mn doped ZnAgInS/ZnS nanocrystal	NMs	* Snowflake-like MoS_2_@Cu_2_S composite	Ultrasensitive, wide linear range, high selectivity, and good stability.	[159]
Ru@Zr_12_-BPDC nanoplate	Organometallic complexes	TPrA	Ultrasensitive detection; LoD of 0.14 fg/mL.	[160]
InP/ZnS_MPA-MSA_	NMs	Co-reactant-free ECL	PSA detection; LoD 0.3 pg·mL^−1^; protocol simplifies ECL assay procedure and provides an alternative to both annihilation and co-reactant routes.	[161]
NH_2_-Ru@SiO_2_ NPs	NMs	Nitrogen doped graphene quantum dots	LoD: 1 fg mL^−1^; self-enhanced luminophore; emitter and co-reactant simultaneously existed in the same NPs	[162]
Ru@SiO_2_-Au nanocomposite	NMs	TPA	Very sensitive H_2_O_2_ detection; AuPd NPs used to enhance ECL signal; also served as co-reaction accelerator; nanocomposite quenched by the ferrocene.	[163]
Au@SiO_2_ @ Ru(bpy)_3_^2+^ doped silica	Organometallic complexes	TPA	Highly sensitive response to glutathione.	[164]
Ce^3+^-Doped TbPO_4_	NMs	K_2_S_2_O_8_	Mucin1 detection; LoD: 0.5 fg·mL^−1^ study may pave way for further research on novel direct ECL emission of lanthanides	[165]
Lu/MoS_2_ QDs@ZIF-8	NMs	-	Ultrasensitive detection of microRNA-21; efficiency increased more than luminol–H_2_O_2_ system	[166]
CD-COF/combined with a CRISPR/Cas12a	NMs	* S_2_O_8_^2−^/Bu_4_N^+^	2.21 fM; introduction of CRISPR/Cas12a system improved sensitivity of the biosensor.	[167]
Zr-MOF modified bulk boron carbon oxynitride	Organometallic complexes	S_2_O_8_^2−^	0.2 fg mL^−1^; high-efficient and low-cost ECL; ultrasensitive detection of breast cancer	[168]

Abb: Pc: phthalocyanine: BTC: benze-1,3,5-tricarboxylic acid (trimeric acid); MOFs: metal–organic frameworks; CD_P_s: plant leaf-derived carbon dots: BPDC; 4,4′-biphenyldicarboxylate; MPA: 3-mercaptopropionic acid; MSA: mercaptosuccinic acid; TPA: Tri-n-propylamine; Tr-HOFs: triazinyl-based hydrogen bond organic frameworks; COF: covalent organic framework. Note: * Represents novel co-reactant.

### 5.2. Recent Advances in Co-Reactants

In addition to the luminophores, there are also sacrificial co-reactants whose main function is to furnish highly energetic radicals upon the electrochemical reactions [169]. Tri-n-propylamine (TPrA) is an example of a substance utilized as a co-reactant in ECL, which proves to be a sensitive approach for bioanalytical applications. Recent research suggests that TPrA can boost the ECL signal and generate light effectively in organic solvents. Nonetheless, other studies have demonstrated that TPrA’s effectiveness in generating light through ECL luminophores is limited to an aqueous medium [170,171]. Another example of a co-reactant is H_2_O_2_; it is the most dominating co-reactant in several luminol-based ECL systems [135] and luminol (5-amino-2,3-dihydro-1,4-phthalazinedione) is one of the most prevalently used ECL luminophore reagents hence widely employed in ECL based assays, for example, it has extensively been used to determine H_2_O_2_ [172]. Luminol is oxidized by an application of a voltage-inducing luminescence with innately high sensitivity and a wide linear range [173,174]. As a result of the high sensitivity for H_2_O_2,_ the majority of the applications of luminol-H_2_O_2_-based ECL are usually applied in the detection of H_2_O_2_ [175]. Refer to Table 2 for more details on different developed luminophores based on luminol for the detection of H_2_O_2_.

Of note, however, H_2_O_2_ has been reported to be unstable and very sensitive as a result, it undergoes undesirable decomposition reactions with metal ions and reductants which leads to poor selectivity and limits its bioassay applications [176]. In addition, notwithstanding its reported exclusive popularity in ECL, TPrA is associated with high toxicity, volatility, low solubility, and high cost which limits its diverse applications [177]. In recent past years, the pursuit of finding biocompatible ECL co-reactants for Ru(bpy)_3_^2+^—a predominant ECL luminophore—has become a popular subject of research, as it aims to expand the range of applications and improve sensing abilities [178]. Advances in the field of nanotechnology have also aided in the discovery of NMs which can take up the role of novel co-reactants. For example, boron nitride QDs (BNQDs) have also been synthesized and been established to be new and effective co-reactants in comparison to other co-reactants, such as H_2_O_2_, peroxydisulfate, and TPrA [179,180]. Furthermore, graphitic carbon nitride (g-CN) NMs as novel co-reactants also have the capacity to amplify the anodic ECL of Ru(bpy)_3_^2+^ since their surface has intrinsic amine-bearing groups [181]. In place of TPrA, arginine has also been established as an alternate co-reactant for Ru(bpy)_3_^2+^ owing to its low cost, biocompatibility, and non-toxicity [182]. Kitte et al. [183] also reported on as a novel co-reactant thioacetamide (TAA) for Ru(bpy)_3_^2+^ through an anodic route and this system was used to determine TAA with a low LoD of 0.035 μM. To add on, Liangrui and coworkers have just recently established a novel ternary ECL system based on anthocyanin-derived CQDs synthesized from purple potato skins serving as a green anodic co-reactant [184,185]. Refer to Table 1 for some more recently developed co-reactors.

**Table 2:** The comparison of LOD of some newly different developed luminol-based luminophores for analysis of H_2_O_2_.

ECL System	LoD and Notable Observations	Ref
Luminol immobilized on graphite as a paste electrode	0.7 × 10^−8^ μM; two nearly neutral samples spiked with 3% and 6% H_2_O_2_.	[176]
Luminol nitrogen doped GQDs	1 × 10^−8^ M; detection in water samples.	[185]
Luminol-H_2_O_2_; Flow injection analysis system with ECL detection	3.0 × 10^−9^ mol/L LoD equal to the level of CL; human serum samples.	[186]
Luminol based on synchronous dual sensitization of ECL by using TiNTs and platinum black.	66 pM (H_2_O_2_), 22 nM (resveratrol), and 30 nM (dopamine); human serum samples.	[187]
Luminol-based ECL system and fluorine-doped tin oxide making use of electrode modified with Au nanoparticles	8 nM; Living cell samples.	[188]
Luminol-capped AuNP-modified electrode	1 × 10^−7^ mol L^−1^	[189]

### 5.3. Recent Advances in Electrodes

Numerous approaches have been suggested for designing appropriate electrodes and creating intricate interfaces because the electrochemical characteristics of electrode materials greatly impact the ECL efficiency [124]. ECL can utilize various techniques such as probes [190], microscopy [44,84,191,192], and the BE chip device. The BE chip device is well-known for its wireless functionality, making it a popular choice for ECL bioanalysis. These chips are essential for high-throughput analysis [112]. In light of the foregoing, a novel method for detecting breast cancer cells with high sensitivity has also been reported by Hasan et al. [89]. This method is based on a new system that uses aptamers and a closed BE, which is designed to be used in a 3D-printed microchannel. The technique involves ECL and has potential applications in the diagnosis of cancer. Another ECL-based sensor involving the detection of prostate-specific membrane antigens has also been designed. To fabricate this specific sensing device, a transparent electrode was modified by introducing doubly functionalized multi-walled carbon nanotubes that possess amine groups, along with a monoclonal anti-PSMA antibody [193]. In addition, a glassy carbon electrode modified with carbon nanofibers was also employed in the ECl high-sensitive detection of azithromycin [194]. Another novel paper-based closed Au-BE ECL sensing platform was employed for the detection of miRNA-155. In this study, the BE’s cathode underwent a modification process that involved utilizing the DNA (S1)-AuPd NPs that were prepared and hybridized through a chain reaction [195]. Furthermore, Li and co-workers modified the electrode using a silica nanopores membrane for ECL detection of nitroaromatic explosives [196]. The previous sections also delved into the synthesis and functionalization of BEs.

### 5.4. The Chemistry of ECL Luminophores and Their Mechanism

The ECL technique by and large is reliant on the chemistry and luminescence characteristics of these species. The electrode device in an ECL setup induces the chemical reactions of the luminophores [197]. In turn, the efficiency of ECL generation is dependent on the electrode material, i.e., the luminophores. ECL luminophores are crucial to ECL-based biosensors, as they determine the sensitivity, selectivity, and stability of the biosensors [116]. These chemical species undergo electrochemical processes and produce unstable, reactive species that consequently take part in fast electron transfer reactions leading to the generation of highly energetic species that later emit luminescence after relaxation. As previously stated, different types of luminophores exist and as expected, their chemistry differs and so does their emission wavelength, which is a unique characteristic used to identify light-emitting chemical species [152,155].

The generation of radiant energy in ECL typically results from two categories of reaction mechanisms: co-reactant mechanism and annihilation [116]. The mechanism of reaction for an ECL system is influenced by various factors including choice of luminophore, electrolyte characteristics, and direction of potential sweep [198,199]. It should be noted that modern analytical ECL applications are more or less exclusively based on co-reactant ECL since the reactions occur in aqueous solutions that are environmentally benign [178]; so the vast majority of ECL research is focused on utilizing the co-reactant pathway by either oxidizing or reducing both the luminophore and coexisting co-reactant. However, ion annihilation ECL was the basis on which early ECL originated. Ion annihilation essentially involves the production of an excited state resulting from exergonic electron transfer between electrochemically generated and unstable species, often radical ions, electrogenerated from the same ECL material to produce excitons [161]. Conversely, in co-reactant ECL, an electrically produced radical from a sacrificial oxidant or reductant combines with the polaron of the ECL luminophore to form an exciton [200].

Initial studies on the mechanism involving the ECL system have established that three are electron transfer reactions taking place between an oxidized and a reduced species, both of these are produced at an electrode by alternating the electrode potential. The general mechanism of ion annihilation is outlined in Equations (2)–(5) [201]:

A → e^−^ + A^•+^ (oxidation at electrode) (2)

A + e^−^ → A^•−^ (reduction at electrode) (3)

A^•−^ + A^•+^ → A^•^ + A (excited-state formation) (4)

A^•^ → A + hν (light emission) (5)

For instance, the potential of the working electrode is quickly altered between two dissimilar values so as to produce the oxidized species, A^•−^, and reduced one, A^•−^, (as shown in Equations (2) and (3), respectively), that will then react near the electrode surface to form the emissive state, A^•^ (Equation (4)).

**Figure 7:**
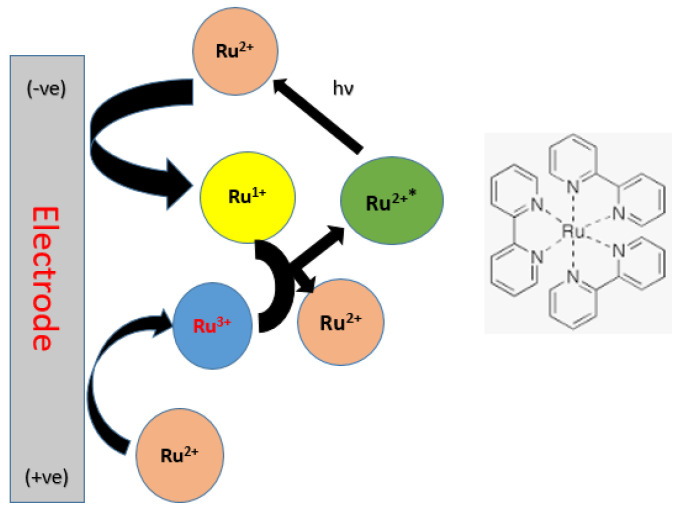
Suggested ECL electrode ion annihilation mechanism and structure of Ru(bpy)_3_^2+^ complex. **Ru^2+^**: Ru(bpy)_3_^2+^; **Ru^3+^**: Ru(bpy)_3_^3+^; Ru^2+^*: Ru(bpy)_3_^2+^*(in an excited state); **Ru^1+^**: Ru(bpy)_3_^+^; * Represents excited chemical spicies.

As earlier noted, Ru(bpy)_3_^2+^ complexes are widely employed in ECL as luminophores. Their mechanisms have been comprehensively studied and well summarized. The following is an illustration of a reported proposed ion annihilation mechanism (Equations (7)–(9)) for Ru(bpy)_3_^2+^ complexes luminophores including its structure in Figure 7. Here, Ru(bpy)_3_^•2+*^ is representative of a molecule in an excited state that emits radiant energy upon relaxation, and hν is a photon of light [202]:

Ru(bpy)_3_^2+^ → e^−^ + Ru(bpy)_3_^3+^ (6)

Ru(bpy)_3_^2+^ + e^−^ → Ru(bpy)_3_^+^ (7)

Ru(bpy)_3_^3+^ + Ru(bpy)_3_^+^ → Ru(bpy)_3_^•2+^ + Ru(bpy)_3_^2+^* (8)

Ru(bpy)_3_^•2+^* → Ru(bpy)_3_^2+^ + hν (9)

Thus far, Si QDs, chalco-genide QDs, and C dots are some of the many kinds of novel QDs that have also been applied as luminophores in the ECL sensors. The general annihilation ECL mechanism involving QDs is presented in reactions (10)–(13) [136]:

QD → QD^•+^ + e^−^ (electrochemically oxidized) (10)

QD + e^−^ → QD^•−^ (electrochemically reduced) (11)

QD^•+^ + QD^−^ → QD^∗^ + QD (annihilation) (12)

QD^∗^ → QD + hν (ECL emission) (13)

**Figure 8:**
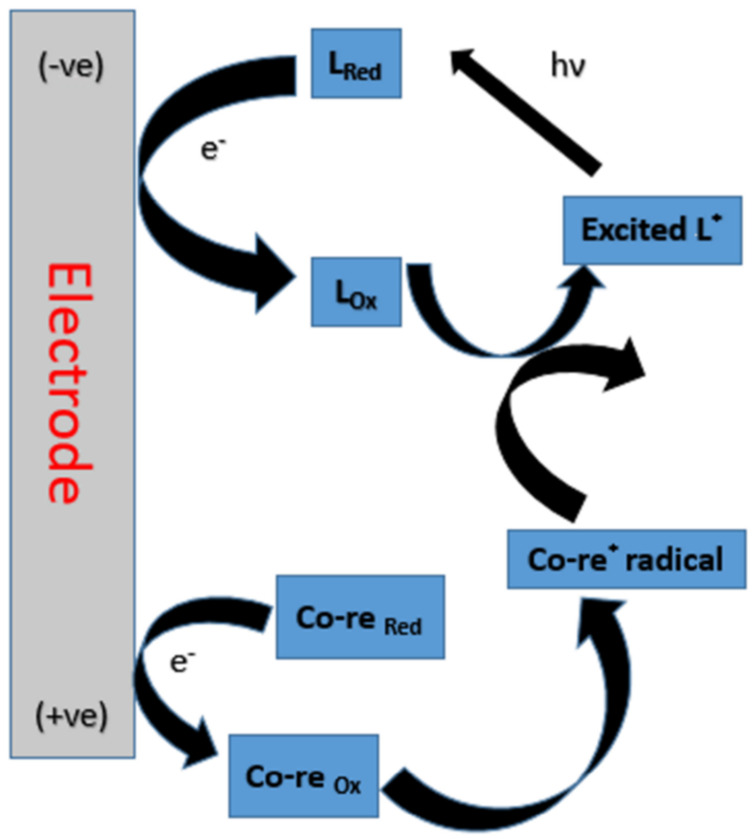
Suggested general ECL electrode co-reacatant mechanism. **Co-re**: Co-reactant; **L**: Luminophore; * Represents excited chemical species.

The co-reaction mechanism of the Ru(bpy)_3_^2+^/TPrA ECL system has also been well established [111,203,204,205,206]. The proposed co-reactant pathway is illustrated in Figure 8. Investigations from these studies indicate that the improvement of the TPrA oxidation current may actually result in an increase in the intensity of ECL. Because DBAE belongs to aliphatic tertiary amines, and its molecule structure is pretty much similar to TPrA, a mechanism similar to Ru(bpy)_3_^2+^/TPrA system can also be suggested to Ru(bpy)_3_^2+^/DBAE system [207]:

Ru(bpy)_3_^2+^ → Ru(bpy)_3_^3+^+ e^−^ (14)

DBAE → [DBAE^•^]^+^ + e^−^ (15)

[DBAE^•^]^+^ → DBAE^•^ + H^+^ (16)

Ru(bpy)_3_^2+^ + DBAE → Ru(bpy)_3_^2+^* + Products (17)

Ru(bpy)_3_^2+^* → Ru(bpy)_3_^2+^ + hν (18)

The DBAE radical cation (DBAE^•2+^) is supposed to lose a proton by oxidation forming the strongly reducing intermediate DBAE^•^ this radical then reduces Ru(bpy)_3_^3+^ to Ru(bpy)_3_^2+^*. In essence, ECL emission is increased by the synchronization of DBAE.

In addition, the co-reaction of the ECL system of the novel Ru(bpy)_3_^3+^/BNQDs ECL pair is presented in Equations (19)–(23) [178]:

Ru(bpy)_3_^2+^ − e^−^ → Ru(bpy)_3_^3+^ (19)

BNQDS-NH − e^−^ → BNQDs-NH^•+^ (20)

BNQDs-NH^•+^ → -H^+^ + BNQDs-N^•^ (21)

u(bpy)_3_^3+^ + BNQDs-N^•^ → Ru(bpy)_3_^2+•^ + product (22)

Ru(bpy)_3_^2+•^ → Ru(bpy)_3_^2+^ + hν (~620 nm) (23)

Various luminophores have been developed for various ECL-based detection of analytes. Below is Table 2, which summarizes the application of various luminophores and compares their limits of detection for H_2_O_2_ as the analyte. H_2_O_2_ has extensive and indispensable applications in food, environmental, and clinical analysis. In addition, at low concentrations, H_2_O_2_ plays a significant function as a regulatory mediator in signaling processes relating to acute diseases [208,209]. It has gained prominence in recent times as a crucial redox signaling molecule in diverse physiological activities and conditions, such as circadian rhythm, inflammation, tissue regeneration, cell proliferation, differentiation, and even the aging process [210]. Thus, its determination in a selection of systems including biological systems is of great relevance.

Nevertheless, in as much as the ECL technology possesses several intriguing advantages as highlighted, it still needs further continuous improvement to meet the ever-growing demands for techniques with even lower detection limits. Coincidentally, advances in other relevant areas of science have turned out to be of invaluable service in this regard, e.g., the development and improvement of nanotechnology have offered potential, practical solutions to further improve limits of detection for ECL and its overall performance because of the flexible controllability nature of NMs which endows them with a variety of valuable, prospective application scenarios including in ECL [211]. The introduction of NMs into ECL-based biosensors has greatly enlarged the applicable pH range and thus generally improved analytical performances of the biosensors [112], for example. It is for this reason that, NMs differing in physical and chemical characteristics such as sizes, morphologies, and inherent chemical components have been adopted in different biosensing applications [212] including in ECL. Since the ECL technique is further disadvantaged by low ECL efficiency associated with some newly developed ECL emitters, there is still a need to develop new emitters and co-reactants to further improve the performance of ECL.

**Scheme 1:**
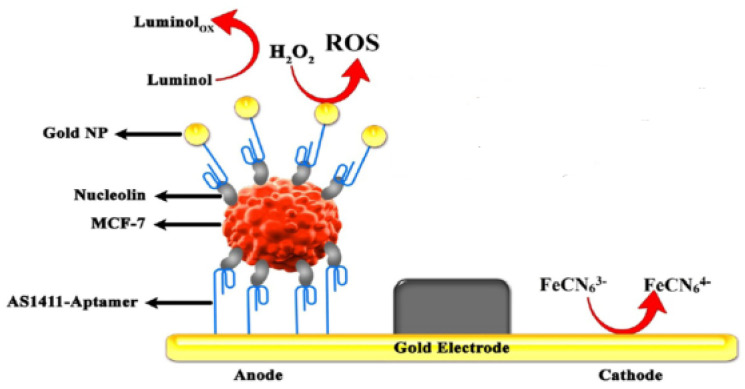
Schematic illustration of the mechanism of electrochemiluminescence detection of human breast cancer cells. Reprinted with permission from [89]. Copyright (2016) Elsevier.

Based on the discussion thus far, the combination of ECL and BPE is one area that has emerged as a potentially powerful tool for biosensing applications. ECL involves the use of luminophores that emit light upon electrochemical stimulation, while BPE involves applying an electric field to the sample, which enhances the mass transport of analytes, improving their detection sensitivity. The advantage of using ECL with BPE lies in their synergistic effect. ECL provides a highly sensitive and selective detection method, while BPE enhances the analyte mass transport, leading to more efficient and sensitive detection from a reduced sample volume. This combination leads to a more sensitive, selective, and reproducible detection than other conventional detection methods. To attain sensitivity and selectivity in ECL-BPE, the cathodic or anodic pole of the BE can be modified. The surface of the BE is modified with a layer of particular capturing chemical species that can selectively bind the analyte. For example, Motaghi et al. modified the electrode with the AS1411 aptamer, a specific aptamer for the nucleolin, and treated it by the secondary aptamer-modified Au-NPs to specifically capture cancer cells. This is illustrated in Scheme 1. Moreover, in this study, BE systems were developed using microchannels fabricated using 3D printing. The aim here was to also minimize the required sample volume [89]. The BE allows for a significant reduction in the sample amount required and also lower power consumption than conventional ECL techniques. The specificity of ECL-BPE can be attained by using special labels or probes that can selectively bind a particular analyte of interest. For instance, at the cathode of the BPE, a sandwich-type cancer cell recognition model using folic acid (FA) and an aptamer as two analyte recognition molecules was developed by Wu and co-workers [213]. In this study, it was shown using control experiments that the sandwich assay specifically collected cancer cells and identified only target cells.

**Figure 9:**
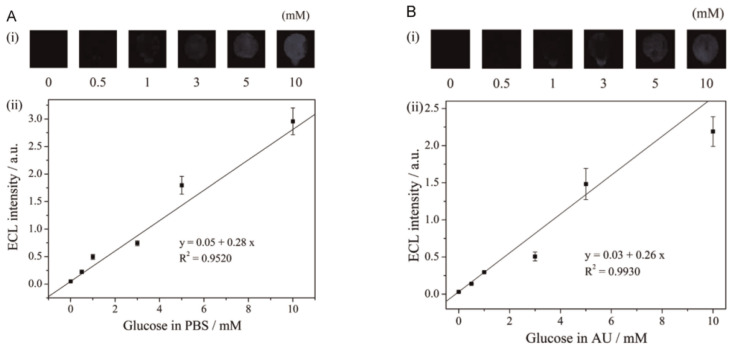
ECL images (i) and intensities (ii) for determination of glucose in pH 7.4 PBS (**A**) and pH 6.0 AU (**B**) samples. Here, the working electrode was modified by 3 μL GOD solution (1.0 U μL1), the concentration of luminol was 2.5 mM, while the scan potential range and scan rate were 0.5–1.0 V, and 100 mV s1, respectively. The error bar represents the standard deviation from five independent measurements taken using three cloth-based devices (each of which contains two sets of ECL units). Reproduced with permission from Ref. [215]. Copyright (2016) Elsevier.

Additionally, electrochemical conditions and parameters such as pH and potential, can be adjusted accordingly to optimize the generation of the target electrochemically active chemical species at either pole of the BE to enhance the specificity of the ECL-BPE technique. In another study, by enhancing the CdS NCs as ECL donors and using the AuNPs as acceptors, an ultra-sensitive ECL immunosensor for selective and sensitive monitoring of p53 protein (a cancer biomarker) using a sandwich approach was designed and fabricated. The immunosensor was prepared by immobilizing the primary capture antibody using a glassy carbon electrode and CdS nanocrystals, creating an immunocomplex between the primary antibody and the p53 protein, and adding the secondary antibody that was conjugated with AuNPs decorated with thiolated graphene oxide [214]. Furthermore, ECL with BPE permits real-time detection and analysis of analytes without the need for labeling or subjecting the sample to other complicated sample preparatory procedures, leading to a reduction in the overall cost and time required for the analysis. This enables the detection of trace levels of analytes in complex matrices, such as biological samples, leading to a better understanding of biological processes and early disease diagnosis. To have a basic comprehension of the transduced ECL signal as a function of the concentration of a target molecule, Figure 9 is referred to. This figure is an illustration of a plot of ECL intensity against the concentration of glucose in a phosphate buffer solution (PBS) and artificial urine (AU) samples. The combination of BEs and ECL has demonstrated the potential for the development of highly sensitive multiplex biosensors. Let us now explore the recent and novel reported applications of BPE-ECL for biosensing in the next section.

## 6. A Critical Review of Recent, Novel Applications of ECL-BPE for Biosensing

**Figure 10:**
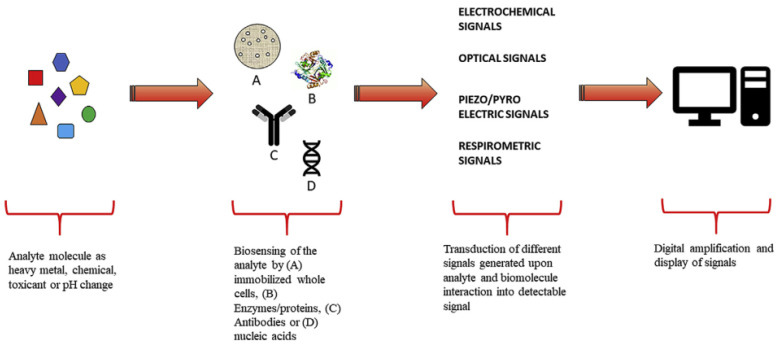
Generic illustration of the signal processing steps in any sensor. Reproduced from Ref. [211]. Copyright (2018) Elsevier.

For any device to function as a biosensor, it should be composed of three crucial components. Forming the first part of any sensor is the biological element which is responsible for recognizing a particular species of interest and generating a response signal in the process. A transducer is the second component that transforms the prior generated signal into a detectable signal. The transducer is the most crucial component of a sensor. The third component is the detector which basically amplifies the signal and processes it and later on displays it on some kind of electronic display system [216]. Biosensors are highly advanced analytical tools that can translate biological responses into electrical signals [217]. When contrasted with conventional techniques, such as ELISA (enzyme-linked immunosorbent assay) or PCR (polymerase chain reaction), biosensors offer many benefits, including affordability, speedy analysis, exceptional sensitivity, precise specificity, and convenient usage [218]. Pertaining to the construction of biosensors, recognition elements that are routinely employed include single and double-stranded DNA, antigens, and antibodies [217,219]. For signal output, there are two types; single-signal output and multiple-output [211]. This biosensing procedure is illustrated in a pictorial form in Figure 10 below.

In the area of clinical chemistry for instance, designing biosensors that are efficient and highly selective for the detection of specific biomolecules for early diagnosis of diseases is a matter of great significance. An array of biosensors for diverse bioanalytes is in use today but noteworthy is that the first biosensor ever to be developed was by Clark and Lyons who invented it for the measurement of glucose in biological samples in 1962 [220]. The rapid development of biosensors has seen the replacement of analytical techniques which are generally characterized to be tedious, costly, and complex to operate [221]. The other notable advantage of biosensors emanates from the fact that it creates a fertile ground to unite and apply a diverse of knowledge ranging from optics, nanoscience, chemistry biology, physics, and materials sciences to electronics [222].

As asserted in the preceding sections, the majority of the studies under BPE focus generally on the electrochemical reaction taking place at the ends of the BEs which in turn rely on other auxiliary optical probes [48,52] such as ECL, for reporting of a signal. Normally, different electrode sets are essential to perform the ECL reaction. It has been suggested that ECL biosensor based on BEs has emerged as a very sensitive, high-throughput, and cost-effective approach [223]. In recent times, the use of BE has allowed the integration of ECL into an electronic sensing device referred to as lab-on-a-chip systems [224]. For instance, BPE-based ECL biosensing is a sensitive detection approach that is successfully utilized in microfluidic chips. In this biosensing platform setup, the system possesses the advantages of high sensitivity, concentration enrichment, low-cost, very portable sensor system, and absence of both a light source and a direct external connection to the electrode [74]. Our research group has also successfully explored the use of a graphite paper-based closed ECL-BPE sensing platform for quantitative analysis of carcinoembryonic antigen biomarker [41]. Table 1 is referred to for more details.

Manz et al. in the year 2001 were the first researchers that introduced and incorporated ECL into BPE where it was ably applied for micellar electro-kinetic chromatographic separation of dichloro-tris(2,2′-bipyridyl)ruthenium(II) hydrate (Ru(bpy)_3_^2+^) and dichloro-tris(1,10-phenanthroline)ruthenium-(II) hydrate (Ru(phen)_3_^2+^)) [37,54]. The reactions involving the co-oxidation of tris(bipyridine) ruthenium(II)/tri-n-propylamine ([Ru(bpy)_3_^2+^]/TPA) and one involving 3-aminophthalhydrazide/hydrogen peroxide (luminol/H_2_O_2_) are among the most effective and broadly employed ECL reactions [215]. Despite ECL reagents having proven incredibly efficient to study Faradaic reactions, it is still a challenge to apply this technique to study non-faradaic reactions [225]. Currently, BPE-ECL biosensing has attracted intense interest and has extensively been applied to invasive sample matrices such as whole fluids, such as whole blood, serum, etc. [77] especially for multiplex assaying (refer to Table 1 for more details).

As stated in the foregoing text, to successfully conduct multiplex assaying using ECL-BPE, the system may exploit a collection of multiple electrodes besides fabrication. This particular electrochemical technique has appealing characteristics, such as the option to drive multiple electrochemical reactions simultaneously on several BEs located in the same electrochemical cell setup. Additionally, in the case of c-BPE, the ability to modify the shape and location of the driving electrodes provides considerable flexibility in designing reaction systems [226]. In this regard, however, there are significant drawbacks that may be noted; firstly the procedure can be deemed quite complex and time-consuming owing to the requirement of several fabrication steps to fabricate multiple electrode systems for suitable multiplex assaying [47,107] and secondly, the cell’s geometry has a substantial impact on the pattern and strength of the potential gradient created on the BE’s surface, and monitoring it is challenging due to the wireless nature of BPE. Interestingly, Villani E. et.al. proposed the utilization of ECL as an electrochemical imaging approach to chart the potential gradient distribution in BE that exhibit diverse geometries [226].

Furthermore, when the paper and cloth-based ECL system is in use, fabrication using a complex and time-consuming screen-printing process has extensively been employed. To counteract these issues, M.L. Bhaiyya and coworkers [227] for the first time developed a laser-induced graphene-based single-electrode system for ECL detection and it was validated. The device demonstrated several advantages, for instance, one-step fabrication, ease of use, portability, and low cost. Overall, the use of ECL with BPE has a significant impact on the development of highly sensitive, selective, and cost-effective biosensors for various applications not only in the analysis of a single analyte but also in multiplex detection. Current sensors are limited to analyzing only one target per measurement. However, in clinical ECL-BPE applications where multiple biomolecules are associated with diseases, single-target assays may be insufficient for accurate diagnosis. BPE devices can control multiple electrodes with a single power supply, making it a competitive option for high-throughput sensing arrays. As a result, diverse sensing strategies have been used to develop multiplex ECL-BP assays [62]. Further research in this area could lead to significant contributions in biosensing and clinical studies for various diseases. Table 3 is a summary of recent and novel applications of closed-BPE-ECL biosensing based on the past 5 years of research.

As can be noted above, ECL-BPE offers several advantages over other electrochemical biosensors. However, there exist several limitations that limit the widespread adoption of this technology in detection purposes. As discussed in the preceding sections, one of the primary limitations of ECL detection using BPE would result from the lack of alternative appropriate electroactive probes. Conventional luminophores such as ruthenium complexes have several limitations, including poor stability and being costly. Regardless, several alternatives to these luminophores have been proposed, including CdSe/ZnS quantum dots, graphene oxide, and other nanomaterials, and research in this area is very currently very active. Another limitation that may be associated with this technique is the intricacy linked to the process of fabricating the electrode. The process of fabrication of BEs is complex as it requires the deposition of two separate metal layers. In an attempt to overcome this challenge, 3D printing of microchannels has been proposed [228].

**Table 3:** An overview of recent and novel applications of closed-BPE-ECL in biosensing.

Sample Matrix/Analyte	ECL System	LoD, Nature of BE and Other Notable Remarks	Ref
Blood/carcinoembryonic antigen and H_2_O_2_	Ru(bpy)_3_^2+^/(NH_4_)_2_C_2_O_4_/H_2_O_2_	5.0 pg mL^−1^ for carcinoembryonic antigen; Pt-graphite paper BE fabricated by depositing Pt NPs.	[41]
Blood/ATP, PSA, AFP, and thrombin	Ru(bpy)_3_^2+^/tripropylamine	Visual detection; Au-ITO hybrid BE; ECL platform enabled sensitive detection and good reproducibility.	[66]
Blood/H_2_O_2_, vitamin B_12_, and vitamin C	Luminol/H_2_O_2_	0.303 μM, 0.109 nM, and 0.96 μM, respectively; laser-induced graphene BE fabricated with polyimide sheet.	[57]
Blood/*S. typhimurium*	([Ir(ppy)_3_] and [Ru(bpy)_3_]^2+^)	10 CFU/mL; ITO BE fabricated with immunomagnetic beads.	[67]
Blood/multi-assaying of cholesterol, glucose, and lactate	PPC−PBA/Fc/Enzyme interface	79 μM for cholesterol, 59 μM for glucose, and 86 μM for lactate; powered ITO electrodes.	[77]
Blood/cholesterol	Luminol/H_2_O_2_	0.12 mM; Potential applications in biomedical, food management and in POCT.	[229]
Blood/PSA	CdTe QDs and luminol	0.5 ng/mL (S/N = 3); Au NRs nanocomposite BE.	[230]
Blood/aflatoxin M1	Luminol-functionalized Ag NPs-decorated graphene oxide	0.01 ng mL^−1^; gold anodic BE coated with magnetic Fe_3_O_4_.	[231]
Blood/miRNA-155 and miRNA-126	CdTe QDs-H_2_ and Au@g-C_3_N_4_ NSs-DNA1/S_2_O_8_^2−^	5.7 and 4.2 fM, respectively; paper-based sensing platform (BE) prepared by wax-printing technology, screen-printing method, and in situ AuNPs.	[76]
Plasma/RASSF1A-methylated DNA and SLC5A8 methylated DNA	Luminol loaded into Fe_3_O_4_@UiO-66	Visual detection; HER on Ru NPs electrodeposited on nitrogen-doped graphene-coated Cu foam and electrooxidation of hydrazine on a polycatechol-modified reduced graphene oxide/pencil graphite electrode used as the BE cathodic and anodic reactions, respectively.	[232]
Serum/glucose, lactate, and choline.	luminol-H_2_O_2_	7.57 μM, 8.25 μM and 43.19 μM, respectively: ITO BEs modified to adapt different enzymes; fabricated with: GOD/MWCNTs/CS (LOD/MWCNTs/CS or COD/MWCNTs/C.	[233]
Cancer cells/adenosine	Ru(bpy)_3_^2+^/TPA	10^−15^ M; ITO BE modified with complementary single-stranded DNA.	[234]
Intracellular/H_2_O_2_ and MCF-7 cancer cells	Luminol/H_2_O_2_	40 cells/mL; AuPd NPs modified BE; wax printing used to fabricate reaction center, and carbon ink-based BE and driving electrodes screen-printed into paper.	[235]
H_2_O_2_	Luminol/H_2_O_2_	0.26 μM; ITO conductive glass as BE.	[236]
Blood/cytokeratin 19 fragments	Luminol/H_2_O_2_ and O_2_	1.89 pg mL^−1^; ITO BE; in situ generation of H_2_O_2_ and O_2_ H_2_O_2_ and O_2_ enhanced luminol ECL intensity.	[237]

Abb: LoD: limit of detection; NPs; nanoparticles: POCT: Point of care testing; ITO: indium tin oxide; PPC-PBA: Phosphorylcholine-phenylbutyric acid; Fc: ferrocene; ATP: adenosine triphosphate; PSA: Prostate-specific antigen; AFP: α-fetoprotein; Ir(ppy)_3_]: iridium (III) complex; (Ru(bpy)_3_^2+^): dichloro-tris(2,2′-bipyridyl)ruthenium(II) hydrate; QDs: quantum dots; NRs: nanorods; NSs: nanosheets; HER: hydrogen evolution reaction; GOD: glucose oxidase; LOD: lactate oxidase; COD: choline oxidase; MWCNTs: multiwalled carbon nanotubes; CS: chitosan; TPA: tripropylamine; CRISPR: clustered regularly interspaced short palindromic repeats.

## 7. Conclusions and Prospects

Taking everything into account, the use of the ECL-BPE technique in biosensing applications has shown great potential for further enhanced selectivity and sensitivity for multiplex assaying. More studies are needed to explore the practical applications of the ECL-BPE sensors in both clinical and environmental settings and to further optimize their sensitivity and selectivity. Future studies could also focus on the development of novel BE materials as well as functionalization strategies that may be able to selectively detect specific analytes in complex samples, as well as the exploration of more diverse applications for the technology. One of the main research gaps observed from this endeavor is the need for further validation and characterization of the biosensors in other complex sample matrices other than blood such as hair, saliva, urine, and environmental samples. For example, the use of ECL-BPE for the detection of drug residues in hair could be an exciting area of research, as it could offer a sensitive and non-invasive tool for drug testing and monitoring. To the best of our knowledge, by the time of writing this review, this application had not been explored, and if it has been, then not as extensively so. The application of the ECL-BPE technique for hair sample analysis can offer significant, distinctive advantages over traditional methods involving sample matrices such as blood. As the hair can store information about drug exposure for months or years [238], hair analysis has become a practical tool in forensics including drug monitoring [239]. Moreover, the most striking feature associated with hair sample matrices is that samples are easily obtainable and conducted so in a non-invasive, and cost-efficient manner.

Further, this review has also looked at different ECL luminophores and how they help address challenges in bioanalytical applications. Since different fields and ECL applications have diverse requirements, new luminophores are highly demanded to address current challenges. Despite ECL advances, some challenges remain and need practical solutions. For example, most ECL luminophores typically emit in a limited spectrum range, making it necessary to develop novel luminophores with tunable colors across the visible spectrum. This will enhance selectivity and enable the detection of multiple analytes [152]. Moreover, molecularly imprinted (MIP)-ECL biosensors are also recommendable for chemical and biochemical analysis due to their enhanced selectivity and sensitivity. However, efforts are ongoing to enhance their sensitivity for trace analysis by using enzymatic amplification or by doping of MIP membranes with NPs such as QDs to amplify the detection signal [240]. In the past 5 years, novel MIP-ECL and enzymatic amplification of ECL-based biosensors have shown improved selectivity and sensitivity for various bioanalytes in blood including HIV-1 gene [241], furosemide [242], enrofloxacin [243], prostate-specific antigen [240], nitrofurazone [244], dopamine [245], kanamycin [246], creatinine, and ethiprole [247].

Resulting from ECL-BPE biosensors possessing several advantages including higher sensitivity, stability, and low limits of detection, its use has emerged as a promising approach for the development of high-performance multiplex biosensors. As such, further research and development of this technique could be crucial for the advancement of clinical diagnosis and forensic science. The use of these methods and related technologies can lead to innovative and cost-effective methods with advantages over traditional, more complex analytical techniques such as HPLC-MS/MS, NMR, etc. Our research group is also yet to extensively explore a number of fundamental aspects and applications of ECL-BPE in the biosensing of a range of biomarkers for diagnostic purposes, for example. It is also recommended to further explore the single electrode electrochemical system in the area of multiplex assaying owing to its inherent superior qualities discussed in the previous sections. While there are still several gaps in the research on the ECL-BPE technique for biosensing, the studies reviewed indicate that this technology has great potential for a wide range of applications in the clinical, environmental, and forensic settings. Further studies in this area could lead to the development of more efficient, sensitive, and selective biosensors with diverse applications.

## Data Availability

Not applicable.
